# Metabonomics-based omics study and atherosclerosis

**DOI:** 10.1186/2043-9113-1-30

**Published:** 2011-10-31

**Authors:** Duo-jiao Wu, Bi-jun Zhu, Xiang-dong Wang

**Affiliations:** 1 Shanghai Key Laboratory of Organ Transplantation, Zhongshan Hospital, Fudan University, 180 Fenglin Rd, Shanghai, China; 2 Biomedical Research Center, Zhongshan Hospital, Fudan University, 180 Fenglin Rd, Shanghai, China

**Keywords:** Metabonomics, metabolomics, atherosclerosis, metabolic disturbances, inflammation

## Abstract

Atherosclerosis results from dyslipidemia and systemic inflammation, associated with the strong metabolism and interaction between diet and disease. Strategies based on the global profiling of metabolism would be important to define the mechanisms involved in pathological alterations. Metabonomics is the quantitative measurement of the dynamic multiparametric metabolic response of living systems to pathophysiological stimuli or genetic modification. Metabonomics has been used in combination with proteomics and transcriptomics as the part of a systems biology description to understand the genome interaction with the development of atherosclerosis. The present review describes the application of metabonomics to explore the potential role of metabolic disturbances and inflammation in the initiation and development of atherosclerosis. Metabonomics-based omics study offers a new potential for biomarker discovery by disentangling the impacts of diet, environment and lifestyle.

## Introduction

Living systems are dynamic and complex, and their behavior may be hard to predict from the properties of individual parts. Systems biology is the strategy of integrating complex data about the interactions in systems of biological components from diverse experimental sources using interdisciplinary tools and personnel [1]. Atherosclerosis is one of the leading causes responsible for cardiovascular morbidity and mortality, a complicated and multifactorial disease associated with genotypes and environmental factors [2,3]. It has been suggested that lipid and inflammatory component play an important role in the pathogenesis of atherosclerosis. Metabonomics is the quantitative measurement of the dynamic multiparametric metabolic response of living systems to pathophysiological stimuli or genetic modification [4]. It is expected that metabonomics will become a more and more important global systems biology tool. Recently metabonomics has been used in conjunction with proteomics and transcriptomics as part of a systems biology description of cardiovascular disease. It utilizes high-throughput approaches to profile large numbers of patients as part of epidemiology studies to understand how the genome interacts with the development of atherosclerosis [5]. Various metabolites have been identified as indicators for a variety of diseases [6,7]. The concentrations of metabolites often vary in response to therapy or disease stage. Furthermore, the metabolites could be used as biomarkers to carry information about the sites and mechanisms of disease. Metabolites have also been used as predictive model for disease risk, individual susceptibility, or as markers of recovery from an illness [8].

Metabolomics requires the employment of efficient analytical tools simultaneously together with bioinformatics. Nevertheless, there is not a single analytical platform nowadays capable of analyzing the full set of metabolites in a biological sample. Metabonomics, and the related field of metabolomics, uses tools such as liquid chromatography-mass spectrometry (LC-MS) or gas chromatography-mass spectrometer (GC-MS)or capillary electrophoresis and nuclear magnetic resonance (NMR) spectroscopy to analyze chemical components [9]. Jeremy K. Nicholson and John C. Lindon [10] said that the distinction between metabonomics and metabolomics is mainly philosophical, rather than technical. The basic principle of relating chemical patterns to biology is same. In practice, the two terms are often used interchangeably. Metabolic disturbances are the key factor in both the initiation and progression of atherosclerosis. There is ample evidence that hypercholesterolemia (that is, elevated plasma levels of low-density lipoprotein (LDL) and very low-density lipoprotein (VLDL) induced by genetic modification or enhanced intake of dietary lipids is a major causative factor in atherogenesis [11,12]. Because samples of biological fluids (usually urine or blood) can be collected fairly easily, the time-dependent fluctuations of metabolites that occur in response to disease, drug effects or other stimuli. By using GC-MS or NMR, metabonomics can easily study these changes in real-time way. And metabonomics cuts through the problems by monitoring the global outcome of all the influential factors for example environmental and lifestyle factor, without making assumptions about the effect of any single contribution to that outcome.

Moreover, to compare with proteomics and transcriptomics, metabonomics is rather rapid and economic [13]. Metabonomics is also a high throughput approach used in a large scale of population epidemic study, while transcriptomic studies can be quite costly and proteomic studies relatively time consuming [13].

### Data handling of metabonomics in atherosclerosis study

Multiple analytical techniques and metabolome database are developed in recent years. Spectral processing and post-experimental data analysis are the major tasks in metabonomics studies. While in data analysis, the Principal Components Analysis (PCA), Hierarchical Cluster Analysis (HCA), Soft Independent Modeling of Class Analogy (SIMCA) and Artificial Neural Network (ANN) are the major techniques. The researchers could select them according to the research destination [14]. The data generated in metabonomics usually consist of measurements performed on subjects under various conditions. Several statistical programs are currently available for analysis of both NMR and mass spectrometry data. The first comprehensive software was developed by the Siuzdak laboratory at The Scripps Research Institute in 2006. It is called XCMS, is freely available, has over 20,000 downloads since its inception in 2006 [15], and is one of the most widely cited mass spectrometry-based metabolomics software programs in scientific literature. Other popular metabolomics programs [16-18] for mass spectral analysis are MZmine, MetAlign, MathDAMP, which also compensate for retention time deviation during sample analysis. Although a high-throughput metabolomics approach to atherosclerosis studies brings many advantages, it also brings a danger of generating false-positive associations due to multiple testing and overfitting of data. Application of traditional statistical approaches (e.g., Bonferroni correction) in this setting tends to levy an insurmountable statistical penalty that can obscure biologically relevant associations. Even newer statistical techniques [19,20], such as advanced resampling methods or control of the false discovery rate, do not adequately address the fundamental problem of how to detect subtle but important changes in multiple variables identified in an "omics" approach.

Meanwhile, there are more and more database, e.g., Small Molecule Pathway Database (SMPDB), LIPID Metabolites And Pathways Strategy (LIPID MAPS), Human Metabolome Database (HMDB) to support metabolomics study [21,22]. HMDB [23] is a Web-based bioinformatic/cheminformatic resource with detailed information about human metabolites and metabolic enzymes. It could be used for fields of study including metabolomics, biochemistry, clinical chemistry, biomarker discovery, medicine, nutrition, and general education. Since its first release in 2007, the HMDB has been used to facilitate the research for nearly 100 published studies in metabolomics, clinical biochemistry and systems biology.

### Metabonomic strategies to study metabolic disturbances and lipotoxicity

**Figure 1:**
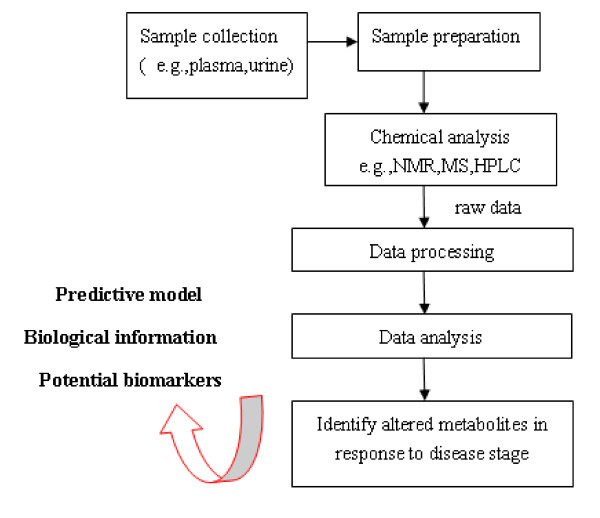
**A "typical" and "simplified" workflow for a metabonomic experiment**. Samples are collected and extracted for the metabolites measurement. By using combination of techniques and data analysis, metabonomics provides information which could be used to identify potential biomarkers, build predictive models for system biology studies.

The global collection of metabolites in a cell or organism is often called the metabolome; this refers to all small molecules that exclude nucleic acids and proteins. There is a new term "Lipidomics" [24], a branch of metabolomics, is a systems-based study of all lipids, the molecules with which they interact, and their function within the cell. Using LC-MS based lipidomics, Clish et al [25] demonstrated altered fatty acid metabolism in 9 weeks apolipoprotein E3-Leiden (ApoE*3) transgenic mice with only mild type I and II atherosclerotic lesions, reflected by an increase in lipid triglycerides and a decrease in lyso-phosphocholine. In an analogous manner, Martin JC, et al [26] gave the hamsters high fat diet to examine the suitability of plasma metabonomics to determine the severity of diet-induced atherosclerosis. They found that VLDL lipids, cholesterol, and N-acetylglycoproteins were the most positively correlated metabolites. These metabolites predicted 89% of atherogenic variability compared to the 60% predicted by total plasma cholesterol alone. Which demonstrates plasma metabonomics may be helpful in disease diagnosis of diet-induced atherogenesis by identifing novel potential disease biomarkers (Figure 1 is a simplified workflow for a typical metabonomic experiment). Furthermore, analyzing different kinds of body fluid simultaneously could provide more description of disease. Zhang F, et al [27] collected plasma and urine samples from the disease and control rats for the metabonomic analysis. 12 metabolites in plasma and 8 endogenous metabolites in urine were identified as potential biomarkers for atherosclerosis. The altered metabolites suggested abnormal metabolism of phenylalanine, tryptophan, bile acids and amino acids.

Metabolic disturbance of atherosclerosis exist not only in circulation, but also could be found in local vessel. Using 2-dimensional gel electrophoresis and mass spectrometry, researchers identified 79 protein species that were altered during various stages of atherogenesis [28]. Simultaneously by using NMR, they found a decline in alanine and a depletion of the adenosine nucleotide pool in vessels of 10-week-old apolipoprotein E-knockout mice [28]. More importantly, the study demonstrated the power of a combined "-omics" platform. These techniques complemented each other and provided a more comprehensive dataset of protein and metabolite changes during atherogenesis and highlights potential associations of immune-inflammatory responses, oxidative stress, and energy metabolism. The study suggested that vascular cells might respond to hyperlipidemia by metabolizing lipids instead of glucose. Increased fatty acid oxidation would exert a negative feedback on the activity of the pyruvate dehydrogenase complex slowing down glucose metabolism, the main source of energy for the vasculature [29,30]. For further elucidation, the researchers observe the effects of attenuating lesion formation. They found it was associated with alterations of reduced form of nicotinamide-adenine dinucleotide phosphate (NADPH) generating malic enzyme, which provides reducing equivalents for lipid synthesis and glutathione recycling, and successful replenishment of the vascular energy pool [28].

Metabonomics also offers a deep insight on the clinical study of atherosclerosis related disease. The plasma of patients with stable carotid atherosclerosis have been fingerprinted with both GC-MS and 1HNMR [31]. 24 metabolites that were significantly modified in the group of atherosclerotic patients and were associated to alterations of the metabolism characteristics of insulin resistance that can be strongly related to the metabolic syndrome. For example, D-glucose, 3-OH-butyrate (3HB) and acetoacetate were increased; citrate, isocitrate, succinate and malate were downregulated. The correlations among the results of GC-MS and 1H NMR fingerprints can provide complementary information and a deeper insight into the patient state. However, clinical investigation is few, while most studies have focused on the pathophysiological study of atherosclerosis on the animal models. Given the current interest in this field, particularly in drug efficacy assessment and lifestyle and diet interventions, there is urgent needs to enhance clinical metabonomics study [13].

### Metabonomic strategies to study inflammation in atherosclerosis

More and more studies in basic and experimental science have illuminated the role of inflammation and the underlying cellular and molecular mechanisms that contribute to atherogenesis. The development of atherosclerosis-induced metabolic perturbations of fatty acids, such as palmitate, stearate, and 1-monolinoleoylglycerol, showed that palmitate significantly contributes to atherosclerosis development via targeting apoptosis and inflammation pathways [32]. Metabolic disturbances in the vasculature stimulate local secretion of inflammatory cytokines. Recent studies implied that the metabolic actions of cytokines such as Interleukin-6 may aim to maintain glucose homeostasis in the smooth muscle cells and contribute to the general adaptation of the vasculature to stress stimuli [33].

**Figure 2:**
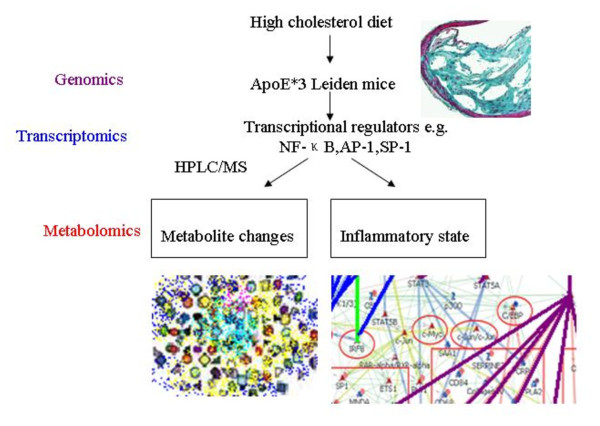
Combined study of transcriptomics and metabolomics in atherosclerosis and liver inflammation induced by high fat diet in ApoE*3 Leiden mice[34]. ApoE*3Leiden mice were treated with high cholesterol diets (HC), scored early atherosclerosis and profiled the pathophysiological state of the liver by using transcriptomics and metabolomics techniques. In HC group, the livers of mice switched from a resilient state to an inflammatory, pro-atherosclerotic state and developed atherosclerosis. HC-evoked changes were regulated by transcriptional master regulators. These regulators control both lipid metabolism and inflammation, and thereby link the two processes.

Given the strong interaction between metabolic disturbances and inflammation, we would expect metabonomics study should hold substantial promise in defining the mechanism involved in this collection of pathologies. Kleemann and co-workers [34] used a combined metabolomic and transcriptomic study of the liver to investigate the inflammatory component of atherosclerosis that originates in this organ (Figure 2). In highest fat diet (HC) group, atherosclerotic lesions of ApoE*3 Leiden mice was proportional to dietary intake of cholesterol, with pro-inflammation being observed in the liver. To verify whether the switch from metabolic adaptation (with low-cholesterol diets treatment, LC) to hepatic inflammatory stress (with HC treatment) is also reflected at the metabolite level, they performed a comprehensive HPLC/MS-based lipidome analysis (measurement in total of about 300 identified di- and triglycerides, phosphatidylcholines, lysophosphatidylcholines, cholesterol esters) on liver tissue of Con/LC/HC groups and corresponding plasma samples. The clusters of the Con and LC groups overlapped partly, demonstrating that the Con and LC groups have a similar intrahepatic lipid pattern. Which indicates that the metabolic adjustments of genes in the LC group were effective and enabled the liver to adjust to moderate dietary stress. The HC cluster has no overlap with the Con group, showing that the switch to a proinflammatory liver gene expression profile is accompanied by development of a new metabolic hepatic state, which differs significantly from the Con group. Furthermore, while the LC induced transcriptional changes that appeared protective, predominately controlled by sterol regulatory element binding protein (SREBP)1 and SREBP2, specific β1 glycoprotein (SP-1), retinoid × receptor (RXR) and peroxisome proliferator activated receptor-α (PPARα), the high-cholesterol diet not only induced inflammation but also altered lipid metabolism, thus linking dyslipidaemia and inflammation in this animal model.

Metabonomics study has also been used to assess anti-inflammatory drug efficiency in atherosclerotic cardiovascular disease such as myocardial infarction (MI). Using metabolomic profiling of the inflammatory lipid mediators, Li N and colleagues [35,36] documented a significant decrease in epoxyeicosatrienoic acids/dihydroxyeicosatrienoic acids ratio in MI model, which predicted a heightened inflammatory state. Treatment with soluble epoxide hydrolase (sEH) inhibitors caused altered pattern of lipid mediators from inflammation towards resolution. Meanwhile, the oxylipin profiling showed a significant parallel to the changes of inflammatory cytokines in the model. Although few studies are available, metabolomics techniques provide evidence for new therapeutic potentials of cardiovascular disease.

## Conclusions

As the field of metabonomics advances, the ways in which metabolites affect atherosclerotic states will become clearer, and prevention and treatment of this process will become more focused. The integration of metabonomics with genetics, proteomics, and transcriptomics would provide a systems biology description of atherosclerotic cardiovascular diseases. A major challenge in the future will be the bioinformatics side of metabonomics. For systems biology, the integration of multi-level Omics profiles (also across species) is considered as central element [37,38]. Traditional statistical methods for the study of static Omics datasets are of limited relevance and new methods are required.

## Competing interests

The authors declare that they have no competing interests.

## Authors' contributions

DJW and BJZ drafted the manuscript. XDW designed and helped to revise the manuscript. All authors read and approved the final manuscript.
